# The Mineral Composition of Bone Marrow, Plasma, Bones and the First Antlers of Farmed Fallow Deer

**DOI:** 10.3390/ani12202764

**Published:** 2022-10-14

**Authors:** Żaneta Steiner-Bogdaszewska, Katarzyna Tajchman, Aleksandra Ukalska-Jaruga, Mariusz Florek, Monika Pecio

**Affiliations:** 1 Institute of Parasitology of the Polish Academy of Sciences, Research Station in Kosewo Górne, 11-700 Mrągowo, Poland; 2 Department of Animal Ethology and Wildlife Management, Faculty of Animal Sciences and Bioeconomy, University of Life Sciences in Lublin, Akademicka 13, 20-950 Lublin, Poland; 3 Department of Soil Science Erosion and Land Protection, Institute of Soil Science and Plant Cultivation, State Research Institute, Czartoryskich 8, 24-100 Puławy, Poland; 4 Department of Quality Assessment and Processing of Animal Products, University of Life Sciences in Lublin, Akademicka 13, 20-950 Lublin, Poland

**Keywords:** *Dama dama*, bulk elements, trace elements, toxic elements, ICP-MS, absorption

## Abstract

**Simple Summary:**

Young cervids build their skeleton during the growth of their first antlers, which is why this is a period of great demand for minerals. The mineral compositions of bone marrow, plasma, bones, and antlers are good indicators of changes that occur in the metabolism of farmed fallow deer. The aim of the study was to analyze the concentrations of 21 mineral elements (bulk elements: Ca, P, Mg, K, Na; trace elements: Li, Cr, Mn, Co, Cu, Zn, Se, Mo; and toxic elements: Be, Al, As, Cd, Sb, Ba, Pb, Ni) in the aforementioned tissues. The highest content was characterized by bulk elements whose content increased in bone, bone marrow and antlers according to the following series: Ca > P > Na > Mg > K. The plasma was characterized by a different concentration of individual elements in the order of Na > K > Ca > P > Mg. The analysis of trace and toxic elements indicated that higher levels were recorded in bone tissues (in antlers and then in bones). It is worth emphasizing that the research indicates a negative correlation between Ca and Cd and between Ca and Pb and between P and Pb in tissues.

**Abstract:**

An adequate supply of essential nutrients is particularly important during the skeletal growth and development of young deer, especially in males, who build new antlers each year. The aim of the research was to analyze the levels of 21 mineral elements (including the bulk elements: Ca, P, Mg, K, Na; trace elements: Li, Cr, Mn, Co, Cu, Zn, Se, Mo; and toxic elements: Be, Al, As, Cd, Sb, Ba, Pb, Ni) in the bone marrow, plasma, bones, and first antlers of farmed fallow deer (*Dama dama*). The mineral compositions of tissues were analyzed using inductively coupled plasma mass spectrometry. Higher concentrations of Ca, P, Mg, Cr, Zn, Se, Al, Ba and Ni were found in bone marrow than in plasma. The highest concentrations of Ca, P and Ba were recorded in fallow deer bone, while the highest concentrations of Mg, K, Na, Li, Cr, Mn, Co, Cu, Zn, Se, Mo, Be, Al, As, Sb, Pb and Ni were found in the antlers. Moreover, the research showed a significant negative relationship between Ca and Cd, and between Ca and Pb, and P and Pb (r_S_ = −0.70, r_S_ = −0.80, and r_S_ = −0.66, respectively; *p* < 0.05) in the tissues.

## 1. Introduction

Metabolic changes indicate that in living organisms, there is a constant exchange of elements necessary for life, which are used to build cells and tissues. Some are periodically removed along with secretions and excretions, while others may be stored in the body. Their reserves are usually activated when there is a dietary deficiency. Such a situation may occur during the antler growth period in cervids because this tissue grows fast and requires a substantial mineral transfer from the skeleton through the bloodstream into the antlers during its growth. Adaptive bone tissue remodeling through continuous biomineralization takes place throughout the deer’s life, in contrast to the antlers, which are shed every year [1]. Moreover, the growth of young cervids is the fastest between birth and the first year of life, whereas the subsequent four years are characterized by a lower growth rate [2]. The first antler grows at an average rate of 1.95 ± 0.05 cm per week and reaches maturity after 14 weeks [3]. The young male’s conditions in the early months of life have important effects on its final size and fitness. However, the antlers, which are a secondary sex feature present in cervid males (with the exception of reindeer), especially their size, are important in the establishment of the hierarchy and position of the male and its attractiveness to females [4].

Nutrition that fully meets the needs of young animals, and thus the appropriate concentrations of trace elements and bulk elements and their proportions in tissues and body fluids, determines the proper growth and development of their system. Nutrients transported by the circulatory system are built into the skeletal system, including the antlers of cervids. Nevertheless, blood is the main and first indicator of changes occurring in the organism because it has a transport function and ensures communication between various body systems and thus undergoes constant changes [5,6,7]. The bone marrow of young animals consists largely of hematopoietically active tissue (from immature cells which can develop into all types of blood cells), with relatively little fat. In these animals, all cells of the body, especially blood, are formed primarily in the bone marrow and are then distributed in the blood throughout the body [8]; however, blood is not an indicator of an adequate supply of all minerals, only showing their content in the moment. Moreover, haematopoiesis is a process that is highly consuming due to the rapid turnover of the haematopoietic system and consequent demand for nutrients and dependence on highly specialised bone marrow. It is well established that micronutrients are relevant to blood cell production, although some of the mechanisms for how trace elements modulate haematopoiesis remain unknown [9]. Thus, the bone marrow is the primary site of blood cell production or hematopoiesis, while hematopoietic tissue is highly proliferative. Billions of cells per kilogram of body weight are produced each day. The hematopoietic system is under exquisite local and systemic control and responds rapidly and predictably to various stimuli [10]. Therefore, we can expect its quick response to unfavorable living conditions related to the deer’s environment.

Various physiological dysfunctions related to deficient, excess, or disturbed homeostasis of elements in the animal organism are caused by biological, genetic, or environmental factors [11]. An adequate supply of bulk elements is particularly important during the growth and development of the young animal’s skeleton [3,6,12]. Micronutrients in ruminants, especially their deficiencies, can lead to serious reproductive problems, impede growth, and cause osteochondrosis [13]. The main factor that may lead to disturbances in the organism’s homeostasis is environmental pollution manifested by the contamination of soil, plants, water, or air that is often associated with biogeochemical conditions [14]. Farmed cervids, unlike wild ones, are not exposed to harmful substances to such a large extent because they cannot move, and they receive feed and water from a controlled environment. They have access to permanent pastures and are fed with properly balanced fodder, especially in winter; therefore, they should be free from toxic substances. Despite this, studies on farmed red deer have shown the accumulation of hazardous substances in their tissues [8].

Examining the mineral composition of bone marrow, plasma, bones, and antlers in cervids provides valuable information about the nutritional status of animals, their health, and their future functioning, especially in farm breeding. The mineral content in the bone marrow and bones of deer was investigated, and it differed because of the maintenance conditions of red deer [15] and also owing to age, which was demonstrated in reindeer [16]. However, fallow deer, which are the most commonly bred deer in Central Europe, have not been studied in this regard so far. Therefore, the aim of the research was to determine and assess the mineral concentration using multivariate analysis of 21 elements, including 5 bulk elements: calcium (Ca), phosphorus (P), magnesium (Mg), potassium (K), sodium (Na); 8 trace elements: lithium (Li), chromium (Cr), manganese (Mn), cobalt (Co), copper (Cu), zinc (Zn), selenium (Se), molybdenum Mo); and 8 minerals with toxicological risk: beryl (Be), aluminum (Al), arsenic (As), cadmium (Cd), antimony (Sb), barium (Ba), lead (Pb), and nickel (Ni) in the bone marrow (BM), plasma (P), bones (B) and the first antlers (A) of young farmed fallow deer (*Dama dama*).

## 2. Materials and Methods

### 2.1. Experimental Design

The study was conducted in 2021 on 6 farmed male fallow deer aged 16 months old according to the scheme presented in our earlier publication [15]. Generally, in the breeding scheme, young stags are mainly culled as the hinds are intended for herd reconstruction; therefore, the analysis included studies of male subjects. The animals were bred in the Research Station of the Institute of Parasitology, Polish Academy of Sciences, Kosewo Górne (Region of Warmia and Mazury; Poland; 53°48′ N; 21°23′ E) in compliance with all quality standards and the required permits. The feeding system comprised the use of rotary grazing in pasture plots with an area and density recommended by DEFRA [17], FEDFA [18], and Mattiello [19]. The research was focused on the fawns born in a natural way during the grazing period, lasting from April to November in Poland. The fawns were fed by doe milk and then vegetation from the pastures. In the winter period (from December to March), the animals were fed ad libitum with grass haylage or hay with a moderate nutritional value. Each animal ingested on average 260 g/d of a mixture comprising 70% crushed oats, 15% rapeseed concentrate (containing 33% crude protein; Eko-pasz), and 15% soybean concentrate (with 45% crude protein content; Eko-pasz) and Josera Phosphoreimer multi-ingredient licks (Josera, Nowy Tomyśl, Poland, Table S1).

### 2.2. Sampling

Before slaughter, the body weights of the animals were precisely measured by MP 800 sensors coupled with a Tru-test DR 3000 weight reader with an accuracy of ±1%. The body mass of each animal ranged from 38.0 to 46.0 kg with an average value of 41.92 kg (SD = 3.08 kg).

Blood was collected from the external jugular vein (*vena jugularis externa*) at a volume of 5 mL into heparinized vacuum tubes. The biochemical parameters were analyzed in blood plasma obtained by centrifugation of sampled blood at 3000 rpm for 10 min in a laboratory centrifuge (MPW-350R, MPW Medical Instruments, Warsaw, Poland) at 4 °C.

The metatarsal bone (*ossa metatarsalia*) as well as antlers (completely ossified) were collected from the animals directly on the day of slaughter. The metatarsal bone was dissected by separating the skin, muscles, and tendons with a stainless-steel knife. Subsequently, the fresh bones were appropriately opened using a dental drill, and bone marrow was selected and frozen at −80 °C. Surgical instruments free of the possibility of secondary contamination of the samples with the tested elements were used in the preparation process.

### 2.3. Mineral Content Analysis

Chemical analyzes of element determinations were performed in accordance with the descriptions contained in previous publications [6,7,8,14]. Briefly, the mineral concentrations in the tissues of fallow deer were determined by the aqua regia digestion (sample weight of 0.5 g) with a microwave digestion (Mars Xpress from CEM Corp., Matthews, NC, United States). The obtained extracts were analyzed by inductively coupled plasma mass spectrometry (Agilent quadrupole 7500CE ICP-MS). Internal standards such as 1 mg/L of 45Sc, 89Y and 159Tb were used for the elimination of the matrix effect and to ensure the long-term stability of samples. Moreover, in each sequence of samples, a blank test and the internal reference material were included in analyses for quality control of the analytical process. Among the validation parameters, the recovery for trace elements ranged from 90% to 97%, while the precision of the method defined as a relative standard deviation (RSD) was <3%. The limits of detection were defined at the level from 0.007 to 0.099 mg/kg depending on the determined element.

### 2.4. Statistical Analysis

Statistica ver. 13 (TIBCO Software Inc., Palo Alto, CA, USA) was used to statistically process the obtained raw data. Parametric and non-parametric tests of data analysis were used to evaluate the interplay of variables according to the result of the assumption of normality. The results were expressed as the mean value and standard deviation of the variables, and statistical differences between tissues (plasma, bone marrow, bone, and antler) were tested using Kruskal–Wallis ANOVA on ranks (Kruskal–Wallis H test). Spearman’s rank correlation analysis was used to assess the relationship (r_S_) between individual elements and the content of the selected element in different tissues. Principal component analysis (PCA) was applied to visualize the dataset and demonstrate the relationships among the elements (variables) present in all tissues studied. The Kaiser criterion (eigenvalue of component > 1) was used as a criterion for selecting the number of principal components. P-values were identified as statistically significant at *p* < 0.05.

## 3. Results

Table 1 shows the mean concentration of 21 bulk elements, trace elements and toxic elements in the examined tissues from the bone marrow, plasma, bone, and antlers of farmed fallow deer. As expected, among all analyzed bulk elements, the highest levels of Ca and P were found in bones and antlers, while the highest Mg and Na levels and the highest Ca:P ratio were found in antlers and bones. In general, plasma contained the lowest amounts of Ca, P and Mg, while K, Na, and the lowest Ca:P ratio were found in bone marrow.

The highest concentration of all essential trace elements marked in the research was found in the antlers (Table 1). Despite obvious numerical differences, the results of the Kruskal–Wallis H test did not confirm significant differences in their content in bones (except for Se), and comparable levels in hard tissues were obtained only for Zn (bones 69.79 mg/kg vs. antlers 78.60 mg/kg). Very low concentrations or <LOD values in soft tissues were obtained for Co, Mo, Li and Cr. In general, the antlers accumulated most of the toxic elements, especially Al and Ni, and had levels of Ba and As similar to bones. Antlers also contained the most Be, Cd, Sb and Pb, the content of which in the remaining tissues was negligible or below the detection level.

The relationships among the concentrations of individual elements in the examined tissues were also assessed (Table 1). There was a significant positive relationship between the concentrations of P in the bones and antlers of animals (r_S_ = 0.83, *p* < 0.05), and a negative significant relationship between the concentrations of Cu in plasma and antlers (r_S_ = −0.89, *p* < 0.05); Se in bone marrow and bones (r_S_ = −0.89, *p* < 0.05) and in plasma and antlers (r_S_ = −0.93, *p* < 0.05); Al in bone marrow and antlers (r_S_ = −0.89, P < 0.05); and Ba in bone marrow and plasma (r_S_ = −0.94, *p* < 0.05) (Table 1).

The Spearman correlation coefficients indicate the interdependencies between the examined elements (Table 2). When analyzing the relationships among bulk elements, positive, strong and very strong relationships were detected among all elements in this group (0.58 ≤ r_S_ ≤ 0.95, *p* < 0.05) except for K. Trace elements were also strongly positively correlated with each other (0.45 ≤ r_S_ ≤ 0.88, *p* < 0.05), and the only exception in this group was Se, for which there was a strong relationship with Cr (r_S_ = 0.70, *p* < 0.05) and a weaker one with Zn (r_S_ = 0.46, *p* < 0.05). Positive relationships were also found among toxic elements (0.50 ≤ r_S_ ≤ 0.94, *p* < 0.05), with the only negative correlations found between Ba and Cd and Pb (−0.49 ≤ r_S_ ≤ −0.50). Considering the correlations of elements from the group of bulk and trace elements, the advantage of positive and strong compounds was shown, especially in the case of Mg and all trace elements (0.54 ≤ r_S_ ≤ 0.84, *p* < 0.05), Na except for Se (0.63 ≤ r_S_ ≤ 0.92, *p* < 0.05) and K except for Se and Zn (0.54 ≤ r_S_ ≤ 0.87, *p* < 0.05). Among the important bulk elements, Ca and P were strongly positively correlated only with Cu (r_S_ = 0.56 and r_S_ = 0.57, *p* < 0.05) and very strongly correlated with Zn (r_S_ = 0.86 and r_S_ = 0.88, *p* < 0.05) while strongly negatively correlated with Co (r_S_ = −0.74 and r_S_ = −0.77, *p* < 0.05). A negative correlation was also found for P and Li (r_S_ = −0.69, *p* < 0.05). Similarly, the significant positive correlations were observed for all toxic elements and selected bulk elements such as Mg (0.54 ≤ r_S_ ≤ 0.89, except Cd, Pb and Ni), Na (0.60 ≤ r_S_ ≤ 0.92, except Se and Cd), and K (0.51 ≤ r_S_ ≤ 0.88, except Ca, P, Mg, Zn, Se, Sb and Ba). In turn, Ca and P were positively strongly correlated with Al and As (0.58 ≤ r_S_ ≤ 0.66, *p* < 0.05) and very strongly correlated with Sb and Ba (0.74 ≤ r_S_ ≤ 0.96, *p* < 0.05). Negative and strong relationships were found for Ca and Cd (r_S_ = −0.70, *p* < 0.05) and Pb (r_S_ = −0.80, *p* < 0.05), as well as for P and Pb (r_S_ = −0.66, *p* < 0.05). Analyzing the correlations of trace elements and toxic elements, strong and significant relationships of Al and As were found with all (except Li) trace elements (0.58 ≤ r_S_ ≤ 0.84, *p* < 0.05). Similar strong relationships were found for Pb and trace elements (except Li and Zn) (0.66 ≤ r_S_ ≤ 0.78, *p* < 0.05) and very strong correlations for Ni and trace elements (except Li, Zn and Se) (0.74 ≤ r_S_ ≤ 0.87, *p* < 0.05). Cd was positively correlated with Cr, Mn, and Co (0.62 ≤ r_S_ ≤ 0.65, *p* < 0.05); Sb was positively correlated with Mn, Cu, Zn, Se, and Mo (0.65 ≤ r_S_ ≤ 0.76, *p* < 0.05); and Ba was positively correlated with Zn and Cu (r_S_ = 0.59 and r_S_ = 0.90, *p* < 0.05). The only negative correlation between elements from these groups was found for Li and Cd (r_S_ = −0.60, *p* < 0.05) (Table 2).

Principal component analysis (PCA) was represented by 13 variables and 24 cases. Three principal components (PCs) with eigenvalues of ≥1 (Kaiser criterion) explained 89.69% of the total variance, with an overwhelming value of PC1 (Table 3).

**Figure 1:**
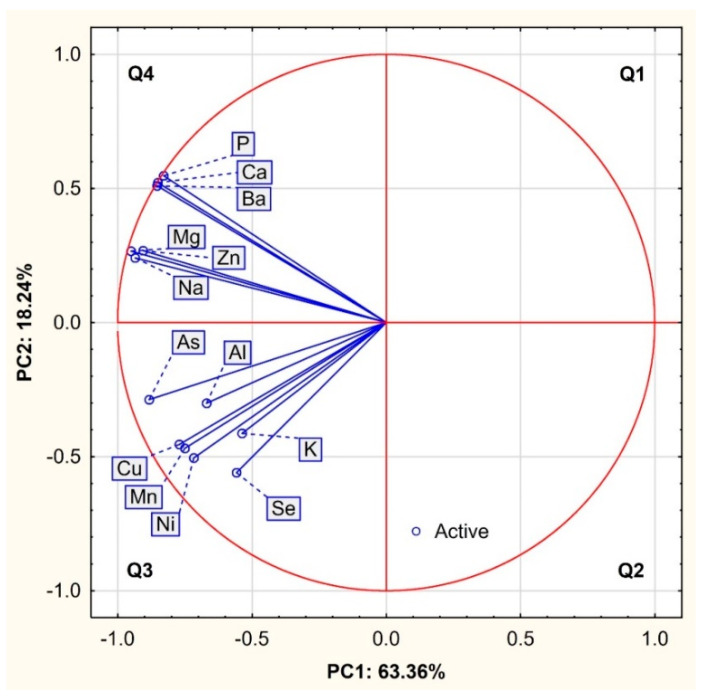
Projection of variables (bulk, trace and toxic elements) in a two-factor plane (PC1 × PC2).

**Figure 2:**
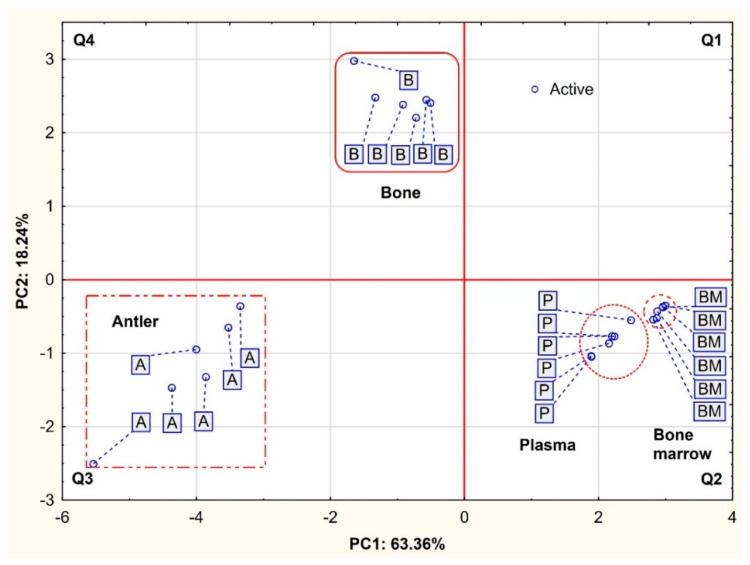
Projection of cases depending on the fallow deer tissues in the two-factor plane (PC1 × PC2).

The projection of variables as a two-factor plane (PC1 × PC2) is presented in Figure 1. Most of the vectors reach the circuit of the plot; thus, all variables are well represented by the first two main components of PCA coordinates. The distance (angle) between vectors confirmed high mutual relationships between bulk and trace elements. The factor loadings matrix details the results of the PCA analysis (Table 3). The first component (PC1) exhibits only negative and high and very high correlations with all variables (Table 3). Based on the loadings and the length of the directional vectors, three groups of elements similar in the properties can be separated according to Figure 2. The first group is composed of Mg (r = −0.95), Na (r = −0.94) and Zn (r = −0.91), and the second group includes Ba (r = −0.85), Ca (r = −0.85) and P (r = −0.83). Both of these groups are located in the upper left quadrant (Q4) defined by negative PC1 and positive PC2 values, indicating different mutual correlations with the presented groups of factors. All other elements are located in the lower left quadrant (Q3), defined by negative values of both principal components (PC1 and PC2). The third group is formed by Cu (r = −0.77), Mn (r = −0.75) and Ni (r = −0.72), which means that the content of Ba, Ca and P is negatively influenced by other elements strongly related to the factors PC1 and PC2. As (r = −0.88) was also very strongly correlated with the first component, while the correlation of Al (r = −0.70) was slightly weaker. In turn, Se was negatively correlated with all three components in a range from −0.44 to −0.56. Only K was most strongly and positively correlated with PC3 (r = 0.66).

Figure 2 presents the projection of cases depending on the animal tissues (B, A, P and BM) in the PC1 × PC2 coordinate system. In general, three groups can be distinguished considering the spatial clustering. The first group located in the lower right quadrant (Q2), i.e., designated by positive PC1 values and negative PC2 values, consisted of both soft tissues (bone marrow and plasma). In contrast, both hard tissues are located on the left side of the graph, with antlers in the Q3 area (negative PC1 and PC2 values) and bones in the Q4 area (negative PC1 and positive PC2 values). This spatial distribution of the tissue samples confirmed their significant variation in mineral composition. This is particularly evident with regard to hard tissue, i.e., antlers and bone.

## 4. Discussion

The research conducted on farmed fallow deer showed a high variability in the levels of elements in the tissues. This was probably caused by the different structures of these tissues, the physiological function, and hence, the different degrees of biotransformation of these elements, as well as the influence of environmental factors [20]. Moreover, mineral concentrations were given in fresh matter and not in dry matter, which vary very strongly between the examined tissues.

### 4.1. Soft Tissues (BM and P)

When comparing soft tissues, higher concentrations of Ca, P, Mg, Cr, Zn, Se, Al, Ba and Ni in bone marrow than in plasma were demonstrated. This research also confirms our previous study on the translocation of minerals within the body and depending on the consumed food and metabolic changes [8,15] due to the fact that blood does not accumulate minerals but only transports them and reflects their amount at a given time. Blood is a tissue with a high renewal rate due to the physiologically short life span of cells in the circulation. The production of these cells is dependent on a highly specialised bone marrow microenvironment, which regulates the quiescence, differentiation and self-renewal of haematopoietic stem cells [9].

Compared to our previous research [15], the contents of Mn and Se were similar to those of farmed red deer (0.027 mg/kg, 0.036 mg/kg, respectively) but higher than those of wild red deer (0.008 g/kg, 0.007 mg/kg, respectively). The contents of Li and Co in the bone marrow of fallow deer, as in both groups of *Cervus elaphus*, were below the detection level [15]. In the studies of Hassan et al. [16] on the bone marrow of reindeer, evidence of Cd, Pb, and As was demonstrated, and in the presented studies in fallow deer, the content of these elements was below the detection level. In the bone marrow of farmed and wild red deer living in the same area [8], higher concentrations of As, Ba, Pb and Ni were found only in wild animals (0.008 mg/kg, 0.969 mg/kg, 0.003 mg/kg, 0.042 mg/kg, respectively), while lower Al levels (2.638 mg/kg) and the same levels of Be, Cd, Sb (<LOD) and Ni (0.014 mg/kg) were found in farmed red deer compared to with fallow deer. The described differences in the concentrations of individual bulk, trace and toxic elements may result from differences between species, but they may also be caused by the different ages of the tested animals and the potential for assimilation of individual elements resulting from individual characteristics.

The Ca:P ratio in fallow deer bone marrow was 1.10 and was lower than that in red deer (1.62) [15]. The optimal concentrations of macro- and micronutrients that should be present in bone marrow in healthy cervids are not known. However, it seems probable that it is beneficial for these organisms to have sufficient concentrations of the analysed substances to form the skeletal system and antlers, which are shed every year, and for the proper course of hematopoiesis.

In previous studies on farmed fallow deer, the level of Ca and P in the plasma was higher in the group without supplements than in the present (2.27 mmol/L, 2.30 mmol/L, respectively) [21]. Kučer et al. [22] and Padilla et al. [23] showed lower concentrations of bulk elements in deer plasma than in the presented studies in fallow deer; similarly, Kuba et al. [24] obtained lower values of Ca, P, and Mg (average: 1.960 mmol/L, 1.836 mmol/L 0.606 mmol/L, respectively) in the plasma of farmed red deer throughout the period of monthly analyses. These differences may result from interspecies differences, but also from the lack of stability of the chemical composition of the plasma, which reacts dynamically to the supply of nutrients and environmental conditions [22]. Minerals are mandatory for the development of effective haematopoiesis, and the absence of these elements can have a deep impact on blood cell formation and/or blood cell functions. Minerals can likely influence haematopoietic functions; therefore, providing proper nutrition should prevent haematopoietic diseases [9].

The Ca:P ratio in the plasma of fallow deer was 1.42 and was higher compared to the results obtained in the group without supplements in the authors’ earlier studies (0.98) [21].

### 4.2. Hard Tissues (B and A)

The highest concentrations of Ca, P and Ba were recorded in fallow deer bone tissue, which is not a surprising result because they are the basic elements of the skeletal system, while Mg, K, Na, Li, Cr, Mn, Co, Cu, Zn, Se, Mo, Be, Al, As, Sb, Pb and Ni were detected in antlers. Minerals such as Co, Cd, and Pb were recorded only in the bones and antlers of animals, Be only in the antlers, and Sb in the plasma and antlers. This confirmed the possibility of some minerals accumulating on the basis of exchange. For example, Mg is required to maintain the correct structure of the calcium phosphate crystals that form the antlers. Moreover, it is irreplaceable in the case of large Ca deficiencies, as it is the divalent cation that is incorporated into the phosphate salts to form a rare form of apatite that is part of the bone mass [25,26,27,28]. This replacement with divalent cations occurs most often when the Ca demand cannot be met by the skeletal system. Mg is probably the basic building material, but it is not the only one because other minerals, including toxic elements, also have such properties, especially in times of critical demand for Ca [24]. Not surprisingly, some toxic elements were only found in the bones and antlers. In addition, their higher concentrations were recorded in the antlers of animals, which confirmed the thesis that they were moved between the tissues, BM, P, and B, and incorporated into the new antlers that grow every year, even though there were no low levels of Ca in the examined tissues. In addition, trace elements easily accumulate in the skeletal system without the possibility of biological degradation.

In the authors’ previous studies, farm fallow deer from the group without supplements showed a lower concentration of Ca (205,000 mg/kg), P (97,000 mg/kg) and Na (5030 mg/kg) and similar Mg (4410 mg/kg) but higher K (460 mg/kg) concentrations in bones compared to the group of animals in the presented study [21]. On the contrary, the levels of Ca (175,000 mg/kg), Mg (4090 mg/kg), and K (570 mg/kg) in the antlers were lower than in the presented group of animals, while the concentration of P (83,500 mg/kg) was similar, and the content of Na (510,800 mg/kg) was higher than in previous studies [21]. This might have been caused by the periodic drought that took place in 2021 and thus affected the different mineral contents in pasture plants available to animals. Chemical analyses of soils and grasses indicated that the greatest mineral deficiencies occur in the dry season [29,30,31]. It was also found that the concentrations of minerals in vegetation change without a predictable trend and may exceed the permissible levels specified for fodder [32,33].

In research conducted on farmed red deer, the contents of Ca (273,469 mg/kg), Mg (4537 mg/kg), and Na (7082 mg/kg) in bones were similar to those in fallow deer but higher in wild red deer [15], while the concentrations of P (110,062 mg/kg), Cr (0.131 mg/kg), Co (0.065 mg/kg), and Zn (63.343 mg/kg) were similar to the results obtained for wild red deer and higher in farm red deer. It was also shown that the level of K (434–551 mg/kg) in the bones of both groups of *Cervus elaphus* was higher compared to fallow deer, but the concentrations of Mn (0.788–1.388 mg/kg) and Se (0.003–0.006 mg/kg) were lower. K is an important bulk element because it contributes to the reduction in Ca loss in urine and to the development of antlers by mobilizing Ca from the skeleton [34]. Manganese, on the other hand, is very poorly absorbed by ruminants, and few studies have suggested that high levels of calcium and phosphorus in the diet may reduce the absorption of this micronutrient [25,35]. It seems that these minerals did not interfere with the absorption of Mn in the test animals. The Se content in the bones was lower than that reported by Olguin et al. [36] (0.42 mg/kg). These differences were likely due the fact that different deer species were analyzed in both studies or Se is mainly stored in muscles rather than in bones, and its deficiency leads to white muscle disease [37]. This may be the reason why bone Se levels do not reflect dietary Se levels.

The mineral composition of bones of one-year-old red deer [38] and fallow deer differed, as lower concentrations of Ca (259,500 mg/kg), Mg (4.220 mg/kg), K (238.9 mg/kg), Zn (41.3 mg/kg), and Mn (1.4 mg/kg) and higher P (122,800 mg/kg) and Ba (317.8 mg/kg) levels in red deer were shown.

The Ca:P ratio in fallow deer bones was 2.62, higher than (2.0) that recommended by Nowicka et al. [39]. A lower Ca:P ratio was usually associated with a higher Ca:Mg ratio in the hard tissues of deer [40].

Landette-Castilejos et al. [41,42] reported lower concentrations of Ca (203,000 mg/kg), Na (5670 mg/kg), Mn (3.52 mg/kg), Cu (0.29 mg/kg), and Zn (55.8 mg/kg) in the antlers of farmed and wild red deer compared to the fallow deer presented in this study, but higher levels of P (98,000 mg/kg), K (549 mg/kg), Co (0.17 mg/kg), and Se (0.71 mg/kg) and a similar Mg (4620 mg/kg) level. Fallow deer antlers, in comparison with *Cervus elaphus corsicanus* [43], contained more Ca (211,700 mg/kg), Mg (4460 mg/kg), K (360 mg/kg) and Na (5550 mg/kg) and less P (9900 mg/kg), Mn (0.47 mg/kg), Co (0.036 mg/kg), Zn (68.73 mg/kg), Se (1.07 mg/kg) and Cu (0.76 mg/kg). The mineral composition of the antlers of three deer species [44], *Cervus duvaceli*, *Axis*, and *Axis porcinus*, was also different from that of fallow deer because the contents of Ca (234,400–220,500 mg/kg), Cu (5.30–8.44 mg/kg), and Zn (32.14–35.81 mg/kg) were lower, P (126,200–108,300 mg/kg), Mg (7300–8000 mg/kg), Co (7.67–9.01 mg/kg), and Cd (1.58–2.18 mg/kg) were higher, and Pb (2.16–2.84 mg/kg) was similar. Additionally, in fallow deer, the Ca:P ratio was higher than in *Cervus duvaceli*, *Axis*, and *Axis porcinus* at the level of 2.76 [44] but slightly lower than in wild and farmed *Cervus elaphus* (2.95) [15]. Moreover, it was shown that the antlers of one-year-old red deer were characterized by a similar concentration of Ca (247,400 mg/kg); higher P (112,200 mg/kg), Ba (320 mg/kg) and Mn (17.1 mg/kg); and lower Mg (428 mg/kg), K (442 mg/kg) and Zn (45.4 mg/kg) [38].

Although the demand for Na is higher during the antler growth period [45], supplementation with Na and other minerals improves rumen buffering and at the same time increases the consumption of minerals [46]. Studies on farmed fallow deer showed a fairly high concentration of Na in hard tissues (bone 7539.4 mg/kg, antler 9204.3 mg/kg). Moreover, it had a significant positive effect on all bulk elements and most trace elements (except Se) in the tested animals, although there is usually an increased demand for this mineral during summer thermal stress and it is usually expelled with sweat [47]. However, it is more dangerous for animals to consume high doses of K in their diet because they inhibit the absorption of Mg and hence raise the possibility of pasture tetany [48], but our studies did not show a significant relationship between these bulk elements. High consumption of Ca and P may also be disadvantageous because they reduce the absorption of Mn [37], but also in this case, no significant relationship between these minerals was demonstrated. Cu deficiency, on the other hand, may occur not only at the level of 5 mg/kg Cu in feed but also with a Mo content above 3–5 mg/kg and Fe above 200 mg/kg [49]. The studied group of animals had access to licks, which could result in higher concentrations of some minerals in their tissues.

As far as we know, this was the first time that the concentration of Li in fallow deer tissues was determined. The effect of lithium on small ruminants has been investigated. Studies have found that 41% of lithium-deficient goats die in the first year of life. Additionally, skin lesions were observed in these animals [50,51]. A deficiency of these trace elements may also have consequences in deer breeding, especially since a significant relationship was shown in our research between Li and most bulk elements (except Ca) and trace elements (except Se).

Chromium is the most abundant mineral in the earth’s crust and can be present in all states of oxidation. Various studies have shown that the primary role of Cr is to activate enzymes and to influence the metabolism of carbohydrates, lipids, and proteins. It is also an integral component of biologically active chromium or the glucose tolerance index, which enhances the action of the important hormone insulin. Insulin will not work effectively in a chromium-deficient diet [52]. This trace element is also necessary for ruminants to counteract stress and increase immunity [53,54]. This confirms its influence on many bulk and trace elements in the tissues of fallow deer.

Cappelli et al. [55] showed that Mn supplementation in red deer increased the mean content of Ca, Na, P, B, Co, Cu, K, Mn, Ni, and Se in the antlers (with a reduction in the Si content), and also had a positive effect on the mechanical properties of the antlers of test animals. Our research also showed a positive relationship between the concentration of Mn and the content of certain minerals such as Mg, K, Na, Li, Cr, Co, Cu, Zn, and Mo in the tissues of fallow deer. However, no relationship was found between Mn and Ca.

The trace elements most important for the grazing of ruminants are Co, Cu, Se, and iodine [56]. Enzootic ataxia [57,58] and osteochondrosis [59,60] are now well-recognized syndromes of copper deficiency in cervids. There is evidence of osteoporosis that is associated with low tissue copper concentrations [61]; the growth of young deer may also be limited [61,62] and antler production may be reduced [63,64]. Another effect of Cu and Zn deficiency may be the reduction in the immune functions of animals [65]. It is worth mentioning that Cu supplementation in deer diet may modify the quality of meat by increasing the protein content [64]. The herd of Norwegian red deer (*Cervus elaphus*) [66] was characterized by insufficient amounts of Cu in the diet, as some animals died from exhaustion, although they had free access to salt licks containing 3000 mg Cu/kg. The herd evaluation showed a poor calf growth rate, low weight of adult hind legs, dull and pale fur, and cases of diarrhea. The studied fallow deer showed two times more Cu in their blood plasma than the described deer before the copper treatment period; moreover, no problems with their hair were observed.

Based on breeding standards, the serum Se concentration for white-tailed deer should range from 0.007 to 0.060 mg/kg [67]. In the tested animals, the concentration of Se in plasma was 0.009 mg/kg and fell within the recommended range. However, studies on farmed fallow deer showed a lower concentration of Se than recommended by Wilson and Grace [68] (Se < 130 nmol/L) for ruminants, similar to the results obtained by Cappelli et al. [55], which also showed a much higher content of this trace elements in deer antlers compared to fallow deer. Wilson and Grace [68] mentioned, however, that if deficiencies appear, it is most often in winter or summer, and the samples from the test animals were taken in late summer after the grazing period. Therefore, most probably, the positive effect of Se in blocking the absorption of toxic minerals (Pb, As) in the tissues of fallow deer was not shown, as indicated in the studies by Łabądź et al. [69] and Vukšić et al. [70]. Moreover, the addition of Se and vitamin E in the cervid diet caused an increase in the number of erythrocytes and level of hemoglobin [71]. The analyses carried out on fallow deer showed a positive correlation of Se with Al, As, Sb and Pb in the examined tissues. Therefore, we cannot confirm the effect of limiting the accumulation of unfavorable Se elements, but it can certainly prevent osteoporosis [72,73] because it has a beneficial effect on the concentration of Mg, Cr, and Zn in the tissues.

In mammals, Mo compounds, especially molybdates, reduce the availability of Cu [74]. Mathieu [75] demonstrated that the effect of molybdenum on animals can be seen at a concentration in the liver of <2 mg/kg regardless of the capture area. The studies on fallow deer showed much lower levels of Mo in the tissues; therefore, it probably did not have a negative effect on the content of other minerals, especially Cu. However, research by Mason et al. [76] and Freudenberger et al. [77] showed that the effect of molybdates on Cu metabolism in cervids may be lower than in other ruminants.

The optimal concentrations of bulk and trace elements that should be present in healthy fallow deer have not been thoroughly investigated. However, it seems likely that it is beneficial for their bodies to have sufficient concentrations of the analyzed substances to form the skeletal system and antlers that are shed every year and to prevent periodic osteoporosis. Unfortunately, the presence of even the smallest amounts of toxic elements may contribute to problems with the skeletal system. This was confirmed by our results obtained in fallow deer, as a significant negative relationship between Ca and Cd, Ca and Pb, and P and Pb was shown. It has been shown that deer bone tissues can serve as environmental bioindicators [78] because accumulate trace elements over years or decades [79]. In humans, for instance, the biological half-life of trace elements in bone tissue lasts up to 30 years, and their content in bones is up to 90% [80]. Moreover, such heavy metals as lead or cadmium are mainly deposited in bone tissues through their interactions with calcium [81].

In fallow deer, as well as in farmed and wild red deer living in the same area, the presence of toxic elements in their tissues was demonstrated: lower concentrations of Sb, Ba, Pb, As and Be, higher Cd concentrations, and similar As and Ba levels [8]. Bone tissues can therefore serve as a reservoir of heavy metals [82]. There are no precise quantitative data on the rate of remodeling of the deer skeletal system; most data concern the movement of minerals and other substances from the bone to the antler as it grows [83]. The significant positive effect of Al and As on most bulk and trace elements (except Li) is noteworthy. Even the smallest amounts of Al and As can have a negative effect on the bone tissues of animals, which was described in more detail in the studies by Tajchman et al. [8], Odstrcil et al. [84], Hu et al. [85], and Priest [86]. This phenomenon may be even more dangerous for deer suffering from cyclical physiological osteoporosis during antler growth [2,87,88,89,90]. Moreover, in vivo studies have shown that the accumulation of Al in the bones lowers the levels of Ca, Mg and P, inhibiting the mineralization process [91].

## 5. Conclusions

The conducted research showed that the bones contain the most Ca, P, Mg, Cr, Zn, Se, Al, Ba and Ni compared to plasma, which results from the nature of the tissues studied and their role in the animal organism. Among trace elements, the Co, Cd, and Pb were only detected in the bones and antlers of animals, while Be was only detected in antlers, and Sb was only detected in plasma and antlers. These results confirmed the possibility of the migration and concentration of minerals in the bone tissue by the exchange processes of divalent cations. Moreover, the higher contents of toxic elements (Be, Al, As, Cd, Sb, Pb and Ni) in the first antlers of young fallow deer compared to the bones indicated a high demand of minerals during the formation of antlers in young growing cervids as well as their high absorption despite the adequate supplementation of Ca.

The research also showed a significant negative correlation between Ca and Cd, Ca and Pb, and P and Pb (r_S_ = −0.70, r_S_ = −0.80, and r_S_ = −0.66, respectively; *p* < 0.05) in the tissues. Despite the use of a properly balanced diet, it is impossible to avoid the adherence of harmful compounds by animals and their incorporation into tissue structures, which may have toxic effects to the body’s health.

## Figures and Tables

**Table 1:** Concentration of elements in fresh matter (mg/kg) depending on tissues of fallow deer.

Elements	Bone Marrow (BM)		Plasma (P)		Bone (B)		Antler (A)		Kruskal–Wallis H Test (3, *N* = 24)	Spearman Rank Correlation Coefficient (r_S_) between Tissues
	M	SD	M	SD	M	SD	M	SD		
Bulk elements										
Ca	951.4 ^ab^	970.4	82.5 ^a^	11.2	285,421.8 ^c^	35,052.9	242,959.3 ^c^	13,771.3	H = 21.600	-
P	691.0 ^ab^	416.3	60.0 ^a^	9.9	109,262.9 ^c^	5943.5	87,852.1 ^bc^	5194.4	H = 21.600	B − A = 0.83 *
Mg	28.4 ^ab^	14.8	16.9 ^a^	2.0	4530.2 ^bc^	610.2	5818.6 ^c^	732.8	H = 20.293	-
K	96.6 ^a^	23.4	773.3 ^b^	94.8	378.7 ^ab^	38.1	832.4 ^b^	123.1	H = 19.867	-
Na	445.0 ^a^	80.5	3072.9 ^ab^	180.4	7539.4 ^bc^	877.8	9204.3 ^c^	992.1	H = 21.117	-
Ca:P	1.1 ^a^	0.6	1.4 ^ab^	0.4	2.6 ^bc^	0.3	2.8 ^c^	0.3	H = 17.760	-
Trace elements										
Li	<LOD	-	0.01 ^a^	0.00	0.42 ^ab^	0.04	2.26 ^b^	1.03	H = 20.236	-
Cr	0.09 ^a^	0.16	<LOD	-	0.15 ^ab^	0.16	3.07 ^b^	2.72	H = 16.120	-
Mn	0.03 ^a^	0.03	0.04 ^ab^	0.50	1.72 ^ab^	0.76	15.30 ^b^	8.96	H = 19.547	-
Co	<LOD	-	<LOD	-	0.06	0.01	0.17	0.13	H = 21.026	-
Cu	0.13 ^a^	0.02	0.61 ^ab^	0.34	2.42 ^b^	1.19	59.05 ^bc^	40.54	H = 20.900	P − A = −0.89 *
Zn	1.23 ^ab^	0.27	0.36 ^a^	0.00	69.79 ^b^	9.98	78.60 ^b^	30.31	H = 19.455	-
Se	0.03 ^ab^	0.00	0.01 ^a^	0.00	0.01 ^a^	0.00	0.06 ^b^	0.02	H = 19.689	BM − B = −0.89 * P − A = −0.93 *
Mo	<LOD	-	0.01	0.04	0.08	0.02	0.38	0.52	H = 18.169	-
Toxic elements										
Be	<LOD	-	<LOD	-	<LOD	-	0.012	0.004	H = 22.395	-
Al	3.700 ^ab^	2.285	0.038 ^a^	0.060	13.550 ^bc^	10.124	89.031 ^c^	48.346	H = 21.239	BM − A = −0.89 *
As	<LOD	-	0.001 ^a^	0.001	0.050 ^b^	0.013	0.183 ^b^	0.027	H = 21.675	-
Cd	<LOD	-	<LOD	-	0.008	0.017	0.026	0.012	H = 20.934	-
Sb	<LOD	-	0.004	0.001	<LOD	-	0.108	0.065	H = 22.256	-
Ba	0.380 ^ab^	0.337	0.039 ^a^	0.035	99.470 ^c^	15.939	85.950 ^bc^	6.182	H = 20.333	BM − P = −0.94 *
Pb	<LOD	-	<LOD	-	0.180	0.116	2.716	1.955	H = 21.934	-
Ni	0.020 ^a^	0.012	<LOD	-	0.260 ^a^	0.061	3.037 ^b^	2.251	H = 21.367	-

M—mean, SD—standard deviation, <LOD—below the limit of detection, * values statistically significant *p* < 0.05, a, b, c differences significant at *p* < 0.05 for multiple comparisons (pairwise).

**Table 2:** The relationship between minerals in the examined tissues of fallow deer in total (*N* = 24).

Analyzed Variable	Bulk Elements				Trace Elements								Toxic Elements						
	P	Mg	K	Na	Li	Cr	Mn	Co	Cu	Zn	Se	Mo	Al	As	Cd	Sb	Ba	Pb	Ni
Ca	0.95 *	0.79 *	−0.08	0.60 *	−0.30	0.03	0.18	−0.74 *	0.56 *	0.86 *	0.19	−0.03	0.58 *	0.64 *	−0.70 *	0.74 *	0.96 *	−0.80 *	−0.01
P	1.00	0.76 *	−0.12	0.8 *	−0.69 *	0.05	0.19	−0.77 *	0.57 *	0.88 *	0.20	−0.13	0.62 *	0.66 *	−0.49	0.79 *	0.93 *	−0.66 *	0.02
Mg		1.00	0.28	0.77 *	0.84 *	0.74 *	0.55 *	0.71 *	0.75 *	0.84 *	0.54 *	0.66 *	0.89 *	0.88 *	0.41	0.74 *	0.83 *	0.52	0.39
K			1.00	0.69 *	0.75 *	0.78 *	0.81 *	0.87 *	0.63 *	0.07	0.09	0.53 *	0.61 *	0.51 *	0.58 *	0.19	−0.02	0.65 *	0.88 *
Na				1.00	0.71 *	0.72 *	0.80 *	0.72 *	0.92 *	0.67 *	0.31	0.63 *	0.83 *	0.83 *	0.47	0.84 *	0.63 *	0.73 *	0.73 *
Li					1.00	0.70 *	0.72 *	0.88 *	0.84 *	0.73 *	0.39	0.69 *	0.52	0.45	0.47	−0.60	−0.30	0.48	0.43
Cr						1.00	0.65 *	0.76 *	0.64 *	0.67 *	0.70 *	0.68 *	0.77 *	0.81 *	0.62 *	−0.03	0.20	0.75 *	0.85 *
Mn							1.00	0.82 *	0.82 *	0.41 *	0.33	0.84 *	0.74 *	0.73 *	0.63 *	0.68 *	0.28	0.76 *	0.87 *
Co								1.00	0.87 *	0.72 *	0.33	0.89 *	0.64 *	0.80 *	0.65 *	−0.03	−0.39	0.66 *	0.85 *
Cu									1.00	0.72 *	0.37	0.80 *	0.84 *	0.83 *	0.35	0.65 *	0.59 *	0.69 *	0.76 *
Zn										1.00	0.46 *	0.45 *	0.77 *	0.81 *	−0.30	0.67 *	0.90 *	−0.05	0.24
Se											1.00	0.23	0.61 *	0.58 *	0.44	0.73 *	0.27	0.78 *	0.25
Mo												1.00	0.58 *	0.81 *	0.53	0.76 *	0.13	0.71 *	0.74 *
Al													1.00	0.94 *	0.65 *	0.64	0.61 *	0.83 *	0.65 *
As														1.00	0.66 *	0.80 *	0.67 *	0.81 *	0.57*
Cd															1.00	0.54	−0.49	0.51	0.50
Sb																1.00	0.70 *	0.54	0.75 *
Ba																	1.00	−0.50	0.06
Pb																		1.00	0.71 *

* values statistically significant *p* < 0.05.

**Table 3:** Correlations between the principal components and the original variables.

Variable	PC1	PC2	PC3
Na	−0.94	0.24	0.23
Mg	−0.95	0.27	−0.00
K	−0.54	−0.41	0.66
Ca	−0.85	0.52	−0.02
P	−0.83	0.55	−0.05
Mn	−0.75	−0.47	−0.17
Ni	−0.72	−0.51	−0.16
Cu	−0.77	−0.45	−0.24
Zn	−0.91	0.27	−0.22
Se	−0.56	−0.56	−0.44
Al	−0.67	−0.30	0.42
As	−0.88	−0.29	0.15
Ba	−0.85	0.51	−0.06
Eigenvalue	8.24	2.37	1.05
% of variance	63.36	18.24	8.09
Cumulative %	63.36	81.60	89.69

## Data Availability

Data is contained within the article or supplementary material.

## Supplementary Materials

The following supporting information can be downloaded at: https://www.mdpi.com/article/10.3390/ani12202764/s1, Table S1: Composition of Josera Phosphoreimer multi-ingredient licks (Josera, Poland).
