# Supplementary material for: The Mineral Composition of Bone Marrow, Plasma, Bones and the First Antlers of Farmed Fallow Deer
Source: Animals (Basel). 2022 Oct 14;12(20):2764. doi: 10.3390/ani12202764 (PMC9597732; doi:10.3390/ani12202764)
Supplement: Supplementary file 1 [file animals-12-02764-s001.zip › animals-1901239-supplementary.pdf]

Table S1. Composition of Josera Phosphoreimer multi-ingredient licks (Josera, Poland)

Components		Content (per 1 kg)
Ca	%	5.00
P	%	10.00
Na	%	7.00
Mg	%	7.50
Ca / P		0.5:1
Vitamin A	j.m.	650, 000.00
Vitamin D3	j.m.	120, 000.00
Vitamin E	mg	1 500.00
Zn (as zinc oxide)	mg	8 000.00
Mn (as manganese chelate of glycine hydrate)	mg	4 000.00
Mn (as manganese (II) oxide)	mg	4 000.00
Cu (as copper sulphate pentahydrate)	mg	1 200.00
I	mg	100.00
Co	mg	22.00
Se (as sodium selenite)	mg	40.00
